# Ageing and digital shopping: Measurement and validation of an innovative framework

**DOI:** 10.1371/journal.pone.0315125

**Published:** 2025-03-19

**Authors:** Michael Olumekor, Sergey N. Polbitsyn, Mohammad Saud Khan, Harman Preet Singh, Ibrahim A. Alhamad

**Affiliations:** 1 Graduate School of Economics and Management, Ural Federal University, Yekaterinburg, Russia; 2 School of Management, Wellington School of Business and Government, Victoria University of Wellington, Wellington, New Zealand; 3 Department of Management and Information Systems, College of Business Administration, University of Ha'il, Ha'il, Kingdom of Saudi Arabia; Sri Sivasubramaniya Nadar College of Engineering, INDIA

## Abstract

Senior citizens are the fastest growing demographic in the world. Amid an intensification of digitalisation across every sector, evidence suggests older people are slow to adopt and use many online tools and services. Moreover, despite studies showing differences in the online behaviour of older people compared to the rest of the population, established models specifically dedicated to explaining their behaviour have remained limited. Therefore, based on components of UTAUT, we propose a new conceptual model that specifically focuses on senior citizens. We introduce four new constructs: health needs, place of settlement (rural/urban), perceived trust, and perceived risk. Data were collected from 320 seniors in Russia and a structural equation modelling was used for data analysis. With a cumulative variance of 86%, the test and validation results demonstrate that our proposed model provides a better explanation of older people’s online shopping behaviour than the original UTAUT model. This model provides an important framework for future studies on the digital shopping behaviours of seniors.

## Introduction

The move towards economic digitalisation has intensified in almost every part of the world. Electronic commerce has become important to businesses, consumers, and the overall macro-economy [1]. It also constitutes a crucial part of the innovative practises of companies because a growing majority of them, including small and microbusinesses, now incorporate some form of online presence into their business models. This includes the use of social media, websites, and mobile applications, among others. However, online commerce is not merely an economic phenomenon. It dominates almost every aspect of social life. Today, a large part of the entertainment products consumed by people, including music and videos, are distributed online. Other products of online commerce connect the social fabric of societies. For example, a majority of social communication in many countries are now largely conducted through online tools such as messenger apps, emails, and social media. Politics and news have also become largely dependent on online technologies, and online tools have become increasingly influential in shaping public opinion [2]. In addition, in many parts of the world, there has been a rise in the acceptance of online education, with many university degrees now being taught exclusively online [3]. Moreover, public services are also increasingly being delivered online [4]. These facts have given rise to the need for studying the digital shopping behaviour of people.

However, while the data on the shopping behaviour and trends of online consumers are clear, most studies have concentrated on middle-aged and younger adults [5–7]. As such, there are very limited studies on the online consumer behaviour of senior citizens, who are also referred to as older adults/ older people in this research. Based on the definition of the United Nations [8], we define seniors as people above the age of 60. The reason for the paucity of studies is due to a number of factors, such as challenges in data collection among older people [9,10]. In addition, resources from online markets and services are also often directed towards attracting younger adults, thereby directly or indirectly disregarding older people. This is particularly surprising because the older population is the fastest growing in the world and is projected to double in size in the future [11]. In many developed and developing countries, older people are among the most financially stable and wealthy [12]. Nevertheless, some recent evidence shows a rise in the acceptance of digital shopping among older people, particularly during the heights of the COVID-19 pandemic [13,14]. The lockdowns imposed to limit the spread of the virus and the susceptibility of senior citizens, pushed many of them to buy and use online products and services, often for the first time.

Furthermore, despite studies showing differences in the motivations, barriers, intentions, and challenges of older adults compared to younger ones [15–17]; models exclusive to the digital shopping behaviour of older people have received scarce attention. Therefore, the main goal of this paper is to provide an empirically grounded understanding of the main drivers and inhibitors of digital shopping use and adoption among seniors. To achieve this, we developed a new conceptual model, building on the Unified Theory of Acceptance and Use of Technology (UTAUT) [18]. Our model extends UTAUT with four new constructs that we argue provide a critically important context for older people. The four new constructs are the health needs of older people, their place of residence, and their perception of trust and risk in online shopping. The dedication of this study to older people makes it different from previous studies that have included other demographic groups alongside older people [19–21]. We tested and validated the conceptual model using a survey of 320 older people in Russia, a country that has remained under-researched in e-commerce studies [22,23]. The results revealed a more accurate analysis of older people’s behaviour than previous versions of UTAUT. The co-variance based structural equation modelling was used for data analysis [24].

In the next section we present the conceptual background of the study. The background includes an extensive discussion of previous literature to develop the constructs and hypotheses of the study. Then, the data, sampling strategy, methods, and empirical approach are outlined. Subsequently, the results, including the model testing and validation, are presented in a series of tables and graphs, followed by a discussion of the findings in relation to prior research. Finally, the limitations of the research are outlined amid recommendations to inspire future research on the topic.

### Theoretical background and hypotheses development

**Fig 1:**
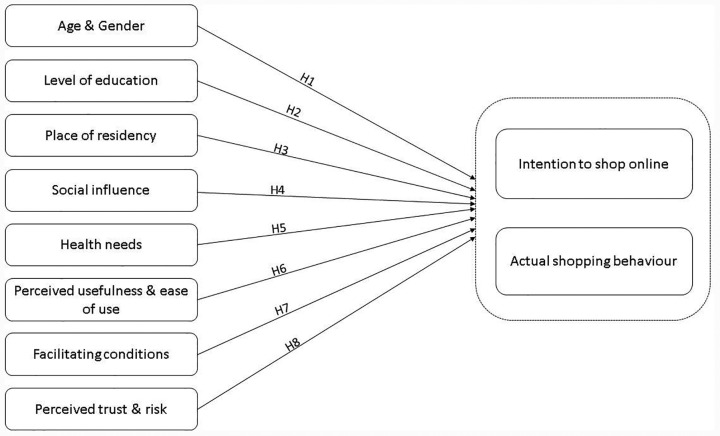
Conceptual model of the study.

The conceptual framework of this research is based on an adaptation of the UTAUT model [18]. Our conceptual model is presented in Fig 1, and a detailed explanation of each construct is provided in the following subsections.

### Age and gender

Age has long been studied as a determining factor of people’s willingness to use technology-related products. However, it became more established when the UTAUT model was introduced [18]. The model argued that age is an important moderating factor that influences people’s behaviour and adoption of online technology. Research from all parts of the world has confirmed this. For instance, a survey in England investigating the acceptance of e-learning technology found that age is an important determiner of user behaviour [25]. Another study in the United Kingdom on online travel reviews and user generated content also found that age moderated the perceived ease of use of online reviews for travellers [15]. Similarly, work examining the usage of e-commerce technology in Indonesia and a meta-analysis of mobile health usage reached similar conclusions [17,26]. Age is a particularly important factor for older people because older age is associated with challenges in processing complex stimuli and paying attention to information [18]. Even among older demographics, age can often be a crucial determinant of decisions to use online tools [27]. In addition to age, research has shown that gender impacts the online behaviour of people. For decades, research on the usage of computers and IT technology has revealed a difference between men and women. One of the leading studies on this issue was a meta-analysis by Whitley, which confirmed the gender variation in the use of computer-related products [28]. Importantly, studies have also shown a difference in the motivations for using technology-related products and services between men and women [18,29]. However, studies have contested the precise nature of this difference. For example, men are more frequent users of online gaming, while women engage more with social media and online fashion shopping, among others [30,31]. Also, while earlier studies revealed that men are more interested in shopping online than women [32], more recent studies are increasingly showing the reverse [33,34]. As a result, gender has also become an established factor in most analyses of digital shopping. Studies on online learning and online travel reviews among both older and younger customers have reinforced the connection between gender and online behaviour [15,25]. Research specific to older demographics has highlighted the impact of gender on their online behaviour [35]. Other studies have revealed that the influence of gender increases with age, and gender plays a stronger role in older demographic groups than in younger ones [36].

As a result, we hypothesise:

*H_1(a)_:* *Age negatively influences the intention to shop online and actual shopping behaviour of older adults.*

*H_1(b)_:* *Gender influences the intention to shop online and actual shopping behaviour of older adults and women shop online more often than men.*

### Education

According to Rogers [37], education consists of skills and knowledge levels that influence people’s acceptance of and understanding of online technology. The level of education of people influences their online behaviour. This could be even more important for older people who are not digital natives because they were not born during the period of internet and smartphone proliferation. According to Porter and Donthu [38], people’s education shapes their beliefs about the internet, which in turn influences their behaviour, while Burton-Jones and Hubona [39] believed that people’s education could influence their knowledge of technology-related services. At the heart of the impact of education is the belief from the social-psychology doctrine and the theory of social learning that people’s behaviours are shaped by their feelings and habits, which in turn are influenced by their education and previous experiences [39]. Many studies have demonstrated the impact of education on internet behaviour. For example, previous studies have shown that the level of education and learning impacts how people feel about internet tools and services and influences their level of success in using IT products and services [40]. Moreover, studies specific to older adults have revealed a significant influence of knowledge and education on their online behaviour [9,10,35,41].

As such:

*H_2(a)_:* *Level of education has a positive influence on the intention to shop online and actual shopping behaviour of older adults.*

*H_2(b)_:* *Seniors with higher levels of education shop online more often than those without.*

### Place of residency

For decades, scholars have lauded the potential of the internet to overcome the geographic, economic, and social challenges of rural areas [42]. However, other studies have pointed to the unique challenges facing rural areas, such as connectivity issues and inefficient delivery infrastructure, as reasons why e-commerce and the internet might struggle to overcome these challenges [43]. Nonetheless, there is little consensus in academic literature on the impact of place of residence on digital shopping. While some studies have shown that people living in rural areas are less likely to shop online [44–46], others have shown that due to the relatively large distance to physical stores in rural areas, people living there are more likely to use online shopping services [47].

There remains a research gap regarding the impact of place of residence among older demographics. However, a recent large-scale study of 61,202 older people across 17 European countries revealed that the place of residence of older people was a substantial determiner of their internet behaviour [35]. The results show that seniors living in rural areas are less likely to use the internet than those living in urban areas, regardless of the country’s level of internet diffusion. This result is consistent with previous studies on older people [48]. Therefore, we argue that the influence of the rural-urban divide is likely to be more pronounced among older people and is crucial for a more holistic understanding of their shopping behaviour. Therefore, we hypothesise the following:

*H_3(a)_:* *Place of residence influences the intention to shop online and actual shopping behaviour of older adults.*

*H_3(b)_:* *Older people living in rural areas shop online less often than their urban peers.*

### Social influence

Initially introduced with the theory of reasoned action, social influence is the belief that people closest to a person play an important role in shaping their online choices and actions. Social influence is often measured as subjective norm [49,50]. Social influence is one of the most frequently analysed determinants of online behaviour. It is the perception that most a person’s significant others believe they should or should not engage in the behaviour in question [51]. According to Venkatesh and Davis [29], social influence is important because people often behave in a certain way, regardless of their personal beliefs, if they believe that people close to them think they should. The impact of social influence and subjective norm has been buttressed in studies around the world. For example, in an investigation of the acceptance of instant messaging in China, a study found that social influence is an important determiner [52]. Another research in China on virtual learning also reached a similar conclusion [53]. Additionally, studies on internet usage among academics, and acceptance of mobile payment technology have supported the impact of social norm on internet related behaviour [54,55]. Furthermore, an extensive meta-analysis of subjective norms found them to be a significant influencer of behavioural intention [56]. Studies on social media usage, augmented reality technology, online food delivery services and online learning environments have found that social influence is among the most important determiners of people’s behaviour [57–59]. While the majority of the aforementioned studies have concentrated on younger adults, several studies on seniors have reached similar conclusions [9,27]. Studies have shown that social influence, which can come from close friends, work colleagues, or family, plays an enormous role in the decision of older people to use online services [9,27]. Therefore, as shown in Fig 1, we propose the following hypothesis:

*H_4_:* *Social influence positively influences the intention to shop online and actual shopping behaviour of older adults.*

### Health needs

There is considerable literature on the health challenges faced by older people when engaging in online shopping [60–62]. Studies have demonstrated that ageing can lead to vision, cognitive, and mobility challenges [60,63,64], which can influence the online shopping behaviour of seniors.

With *health needs*, we postulate that seniors might be more willing to shop online if their health condition encourages or necessitates it and be less willing if their health needs limit them from doing so. For example, older people with mobility challenges might experience difficulty visiting physical stores and instead turn to online shopping services [65], whereas those with cognitive challenges might prefer to visit physical stores [21]. Studies have shown that the health and needs of older adults significantly shapes their internet related behaviour [66].

In addition, the behaviour of older people during the COVID-19 pandemic provides strong evidence of the impact of health needs on their shopping behaviour. Many studies during the Covid-19 pandemic showed an increase in digital shopping usage among seniors, as they were more susceptible to the virus [67–69]. Seniors used the internet for a lot of things including product and grocery shopping, often for the first time [13]. Extensive studies of this period show that older people were motivated to buy things online because it benefited their health needs [69,70]. To put it differently, older people shopped online more because of the potential impact of the pandemic on their health if they went to physical stores. As a result, we hypothesise:

*H_5_:* *Seniors shop online more often when shopping apps and websites meet their health needs*.

### Perceived usefulness and perceived ease of use

The idea that perceived usefulness and ease of use could influence the acceptance or usage of IT-related products was popularised by Davis [71]. Davis defined perceived usefulness as “the degree to which a person believes that using a particular system would enhance his or her job performance” and perceived ease of use as “the degree to which a person believes that using a particular system would be free of effort” [71]. Other studies have referred to the same concept using similar terms. For example, Venkatesh et al. [18] introduced performance expectancy and effort expectancy. They defined performance expectancy as the degree to which using a technology product provides benefits to customers and effort expectancy as the degree of ease with using a technology.

Nevertheless, because our study is specific to online shopping and not to overall technology adoption, we define perceived usefulness as the belief among older people that online shopping provides benefits, and perceived ease of use as the belief among older people that they can easily shop online. Studies on digital shopping have found that these two factors influence the shopping attitudes of people [72]. We argue that because seniors are less familiar with digital technologies [10], their perceived ease of shopping online and potential benefits could be even more important for them. This is bolstered by previous studies that have demonstrated the impact of perceived usefulness and ease of use on the overall internet behaviour of seniors [73], and its considerable influence on their online shopping behaviour in particular [20,74]. Therefore, we hypothesise:

*H_6_:* *Perceived usefulness and perceived ease of use positively influence the intention to shop online and actual online shopping behaviour of seniors.*

### Facilitating conditions

According to Venkatesh et al. [18], facilitating condition is the “the degree to which an individual believes that an organizational and technical infrastructure exists to support use of the system”. As such, facilitating conditions consist of an external ecosystem that can either stimulate online usage or discourage it. Examples of facilitating conditions include technological factors such as a good internet connection [75], having a smartphone or computer, and payment security. It can also include organisational factors, such as good user interface and application speed. Furthermore, facilitating conditions can include an individual’s personal resources, such as money and time [76]. Facilitating conditions mean that people are unlikely to shop online if they do not have the money or time to do so. Moreover, in contemporary times, people are less likely to buy things online if they have a poor internet connection or do not possess a smartphone. Studies show that facilitating conditions influence the digital behaviour of people [18], and the behaviour of seniors in particular [77]. In addition, studies specific to the online shopping behaviour have also demonstrated the influence of facilitating conditions [73,74]. Facilitating conditions are important for older people who might require more unique and specific needs to engage more with online shopping websites and applications [74]. Therefore:

*H_7_:* *Facilitating condition has a positive influence on the intention to shop online and actual online shopping behaviour of older adults.*

### Perceived trust and risk

According to Rotter [78], trust is “an expectancy that the promise of an individual or group can be relied upon”. This definition is grounded in the theory of social learning, which argues that people differ in their expectations when promised by other people/organisations of a specific outcome [78,79]. Trust is a crucial part of any transaction or relationship between two or more entities when there is some level of risk or uncertainty [79]. There is inherent risk in online commerce because it usually involves a non-physical relationship or transaction between two or more entities. Because online commerce often involves giving out private information such as name, address, contact information, and credit card information, it requires some degree of trust to be successful. This is among the biggest challenges for older customers who are less trusting of digital platforms. For online transactions, there is also some degree of risk with respect to the authenticity of the product or service, as they cannot be physically examined before they are bought.

Studies have shown that there are different levels of online trust. For example, scholars have argued that initial trust is one of the most important factors defining online consumer behaviour. Initial trust is defined as the level of trust required to complete a transaction with an online person/organisation for the first time [79,80]. This also involves the highest level of risk because the user/customer has limited information and no prior history with a company. For older adults, developing this initial level of trust is a tough challenge because they have spent large parts of their lives physically examining products or services before paying for it. Furthermore, prior studies have established the influence of both perceived trust and perceived risk on the digital shopping behaviour of seniors [21,73,81]. Consequently, we hypothesise:

*H_8(a)_:* *Trust in online shopping platforms has a positive influence on the intention to shop online and actual online shopping behaviour of older adults.*

*H_8(b)_:* *High risk perception negatively influences the intention to shop online and actual shopping behaviour of older adults*.

## Methods

### Data collection and measurement

Table 1 presents the measurement scales used in this study. Before starting data collection, it was necessary to define exactly who qualified as an older person for our research. Due to the different age limits across many countries, we chose to adopt the United Nations definition of older people as those over 60 years of age [8]. Following this, we conducted a comprehensive scoping review of the extant literature to develop the theoretical model (Fig 1) and identify the initial survey questions for this research. Then, a small focus group, comprising nine older people from four separate cities/towns was conducted to test the initial survey questions and screen out unimportant ones. The feedback from this focus group was instrumental in developing the final version of the measurement scale (Table 1) and survey questions. For example, the initial survey included questions on the earnings and income level of respondents, however participants in the focus group expressed some discomfort with revealing exactly how much they earn. Moreover, the pension system of Russia meant a fairly even base level of income for older adults. As a result, the income question was dropped. Questions on education were also modified to reflect the Russian education system, while other questions were either fine-tuned to convey a more accurate understanding of the Russian language or screened out. The English translation of the final 25 questions used for the survey and their respective categories are included as a supporting information. The questions used a 5-point Likert scale, measuring from strongly agree to strongly disagree, except for questions on sociodemographic characteristics. In addition, the question on actual shopping behaviour, measuring how often respondents shopped online, used answers ranging from always to never (S1 File).

**Table 1:** Measurement scales of survey constructs.

Constructs	Abbreviation in this research	Sources
Place of residence	POR	Zhu and Chen (2013)
Social influence	SI	Venkatesh et al. (2012, 2003)
Perceived usefulness	PU	Venkatesh et al. (2012, 2003)
Perceived ease of use	PEOU	Venkatesh et al. (2012, 2003)
Health needs	HN	^*^Ang et al. (2021)
Perceived trust and risk	PTAR	Pavlou and Gefen (2004), Crespo et al. (2009)
Facilitating condition	FC	Venkatesh et al. (2012, 2003)
Intention to shop online (behavioural intention)	ITSO	Venkatesh et al. (2012, 2003)
Actual shopping behaviour	ASB	Venkatesh et al. (2012, 2003)

All constructs are adapted from the original studies to suit an older person context.

* The construct on health needs is based on a significant adaptation of the original questions from the corresponding study.

This study was performed in line with the principles of the Helsinki Declaration. Written informed consent was received from all research participants and ethical approval was obtained from the ethics committee of School of Public Administration and Entrepreneurship in Ural Federal University (protocol number: 66-62/002/06-22).

The survey was conducted in the Sverdlovsk region of Russia between 17/10/2022 and 31/03/2023. Ethical approval was received from the first author’s institution, and written informed consent was received from all survey respondents. Considering that seniors can be classified as a hard-to-reach demographic group, a mixed sampling design, comprised of the snowball sampling method [82] and time-space sampling [83], were used. While both online and paper-based surveys were used, approximately 97% of all responses came via the paper-based survey. Trained survey collectors were sent to several cities, towns, and villages in the Sverdlovsk region of Russia where they visited places frequented by seniors, such as public parks, markets, and social clubs. In addition, social programmes/events run by municipal administrations and private companies, such as fitness, dance, and art activities, were also visited. In total, 363 responses were collected. However, 43 responses were excluded for containing either incomplete responses or multiple answers to the same question. Therefore, 320 responses were included in the analytical part of this research (n =  320). The research analysis was conducted using R.

### Analytical approach

All 320 responses were entered into a Microsoft Excel spreadsheet before they were analysed. The analysis included confirmatory factor analysis (CFA) and Structural Equation Modelling (SEM). The data were tested for normality using the Anderson Darling Test for Normality (ADTN) of $H_{o}:errorsareapproximatelynormallydistributed$ and it showed that the data is non-normal with a ***P value*** < 0.001. This necessitated the use of a robust maximum likelihood (ML) estimation procedure with CFA and Structural Equation Model (SEM).

The goodness-of-fit index is $\chi^{2}$. In the ML model estimation, $\chi^{2}$is calculated as:


$$\chi^{2}=F_{ML}\left(N-1\right)$$

Subsequently, a Comparative Fit Model (CFI) and the Tucker–Lewis Index (TLI) were used to determine the model fit. The CFI evaluates the fit of a user-specified solution in relation to a more restricted, nested baseline model. It is calculated as:


$$CFI=1-max\left[(\chi_{T}^{2}-df_{T}),0\right]/max\left[(\chi_{T}^{2}-df_{T}),(\chi_{B}^{2}-df_{B}),0\right]$$

Where $\chi_{T}^{2}$ is the $\chi^{2}$ of the target model (i.e., the model under evaluation) $df_{T}$ is the df of the target model, and $\chi_{B}^{2}$,$df_{B}$ the baseline model. TLI is defined as:


TLI=χB2dfB−χT2dfT/χB2dfB−1

To determine the model fit, the CFI and TLI values should be greater than 0.8.

A widely used parsimonious correction of model fit is the Root Mean Squared Error of Approximation (RMSEA) [84]. The RMSEA is a population-based index that relies on the non-central ${\text{χ}}^{2}$ distribution which is the distribution of the fitting function. The non-central ${\text{χ}}^{2}$ distribution includes a noncentral parameter (NCP) expressing the degree of model mis-specification. The NCP is estimated as${\text{χ}}^{2}-df$. To foster the conceptual basis for calculating the RMSEA, the NCP was rescaled to the quantity $d=\chi^{2}-df/N-1$. Then, the RMSEA is computed as follows:


RMSEA=SQRTddf

The following section presents the results of this research.

## Results

**Fig 2:**
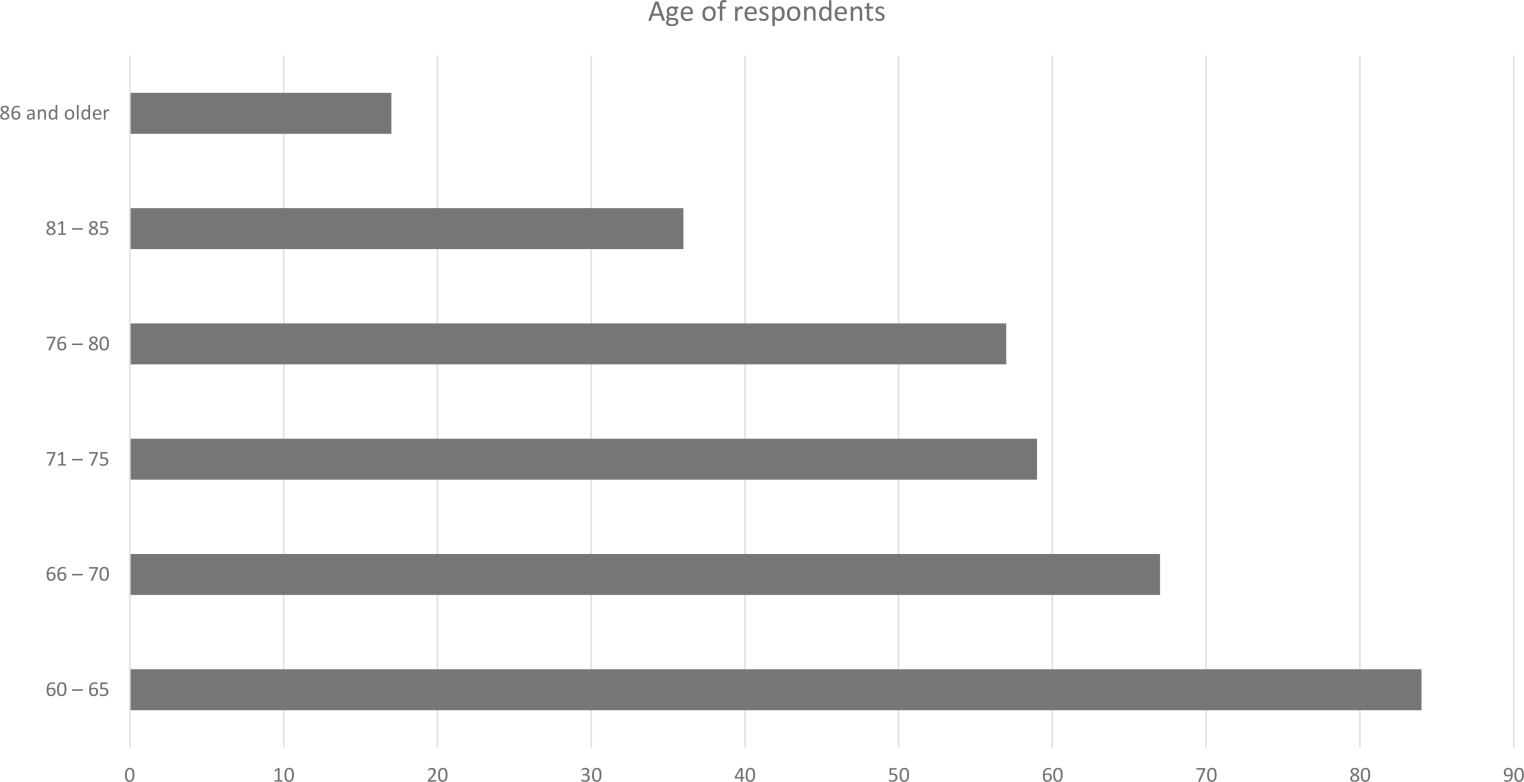
Age of survey respondents.

Table 2 shows the demographic distribution of all survey respondents. The majority of respondents were women (66.3%), while only 33.8% were men. In addition, 39.4% of respondents were university graduates, whereas 5% have postgraduate education. Finally, the distribution of the place of residence of respondents shows that most respondents resided in urban areas (77.2%), compared to rural areas (22.8%). The age distribution of respondents is presented in Fig 2.

**Table 2:** Demographics characteristics of survey respondents.

Variables	Categories	Frequency (%)
Gender	Female	212 (66.3)
	Male	108 (33.8)
	Total	320
Educational Level	Basic education (8th class)	35 (10.9)
	Secondary education (10th class)	29 (9.1)
	Initial vocational education	26 (8.1)
	Secondary specialist education	99 (30.9)
	University education	126 (39.4)
	Postgraduate university education	5 (1.6)
	Total	320
Place of Residence	Rural area	73 (22.8)
	Urban area	247 (77.2)
	Total	320

Fig 2 shows that more respondents belonged to the age group of people between 60 and 65, than any other age groups (23.6%). This is followed by the age groups 66 to 70 (20.9%) and 71 to 75 (18.4%). Seniors aged 86 and older were the fewest people in our sample (5.3%).

Table 3 presents the overall average response below 4 on a 5-point Likert scale questionnaire. It shows some notable findings from this research. First, with a mean of 2.09 for the section on actual shopping behaviour, our results show that most older people do not shop online. Second, with a mean of 2.59 and 2.60 for the question on intention to shop online, older people expressed fairly average intentions to buy things online in the future.

**Table 3:** Descriptive results of the survey.

Items	Mean	Std. Dev.
Perceived Trust and Risk (PTAR)		
PTAR1	2.55	1.58
PTAR2	2.61	1.51
PTAR3	2.48	1.44
PTAR4	4.09	1.25
Social Influence (SI)		
SI1	2.32	1.51
SI2	2.28	1.51
SI3	2.16	1.46
Intention to Shop Online (ITSO)		
ITSO1	2.59	1.56
ITSO2	2.60	1.61
Facilitating Conditions (FC)		
FC1	3.34	1.82
FC2	2.64	1.71
FC3	3.06	1.74
Perceived Usefulness (PU)		
PU1	2.50	1.59
PU2	2.48	1.59
PU3	2.48	1.57
Actual Shopping Behaviour (ASB)		
ASB1	2.09	1.32
Health Needs (HN)		
HN1	2.18	1.54
HN2	2.33	1.50
Perceived Ease of Use (PEOU)		
PEOU1	2.48	1.64
PEOU2	2.45	1.61
PEOU3	2.36	1.58
PEOU4	2.33	1.58

Std. Dev. =  Standard deviation.

### Results of confirmatory factor analysis (CFA)

Factor and confirmatory analyses were carried out to determine the best fit between the observed and items variables. After confirming the non-normality of the dataset, we employed maximum likelihood (ML) robust estimation with selected 8 factors of the items under 0.5 threshold eigenvalues. The reliability of the factors was 98% with RMSEA. In addition, the parallel analysis suggests that the number of factors =  3 and the number of components =  NA. The threshold value for the Eigen value is 1, but in order to account for the various items in the dataset, this analysis gave room for the Eigen value to 0.5, which enabled the choice of 8 factors of the observed variables. Results of the factor identification and cumulative variance are included as a S1 Table. Results of the CFA fitting and testing are presented below:

From Table 4, the model test using the chi-squared test showed that the model is significant with a standard estimate value of 619 and 162 degrees of freedom, with a P value of less than 0.05. Moreover, both measures of CFI and TLI were expected to be greater than 0.8, and the respective values of 0.964 and 0.954 signify the fit of the model. Additionally, from the model above, the RMSEA and SRMR are less than 0.5, further indicating the model fit (Table 4).

**Table 4:** Model fit measures of the structural equation model.

	Standard	Scaled
Test statistics	619.585	377.77
Degree of freedom	162	162
P value (Chi Squared)	0.000	0.000
Robust Comparative Fit Index (CFI)	0.964	
Robust Tucker Lewis Fit Index (TLI)	0.954	
Robust RMSEA	0.082	
SRMR	0.027	

SRMR =  Standardized root mean squared residual.

Table 5 shows that all factor loadings were greater than 0.5 except for PTAR 4. As such, PTAR4 was excluded from the model. Additionally, it was also found from the analysis that factor 5, social influence, has a greater impact than the other factors. Meanwhile, perceived trust and risk have the least influence. The total cumulative variance of the factors was 0.86, which is desirable (S1 Table).

**Table 5:** Results of confirmatory factor analysis.

	Estimate	Std.Err	z-value	P(>|z|)	Std.lv	Std. all	R-squared
PTAR							
PTAR1	1.000	1.462	0.927				0.86
PTAR2	0.982	0.028	35.259	0.000	1.435	0.954	0.91
PTAR3	0.906	0.031	29.085	0.000	1.324	0.918	0.842
SI							
SI1	1.000	1.431	0.949				0.901
SI2	0.998	0.021	46.624	0.000	1.427	0.945	0.893
SI3	0.925	0.026	35.899	0.000	1.323	0.909	0.827
ITSO							
ITSO1	1.000	1.533	0.984				0.967
ITSO2	1.032	0.011	98.211	0.000	1.581	0.986	0.972
FC							
FC1	1.000	1.458	0.801				0.641
FC2	1.081	0.052	20.73	0.000	1.577	0.924	0.854
FC3	0.983	0.042	23.367	0.000	1.434	0.826	0.683
PU							
PU1	1.000	1.505	0.95				0.902
PU2	0.961	0.021	45.348	0.000	1.446	0.914	0.835
PU3	0.964	0.022	43.339	0.000	1.45	0.928	0.861
ASB							
ASB1	1.000	1.318	1.000				1.000
HN							
HN1	1.000	1.261	0.823				0.677
HN2	1.101	0.064	17.073	0.000	1.388	0.925	0.856
PEOU							
PEOU1	1.000	1.541	0.939				0.881
PEOU2	1.012	0.019	54.145	0.000	1.559	0.967	0.935
PEOU3	0.99	0.022	44.69	0.000	1.526	0.968	0.936
PEOU4	0.955	0.026	36.594	0.000	1.47	0.935	0.873

Std.Err =  Standard error. Std.lv indicates that only the latent variables are standardized. Std. all indicates a complete standardized solution whereby both the latent and observed variables are standardized.

### Results of regression analyses

Table 6 presents the calculation of the influence of sociodemographic factors using a generalised linear model. Online shopping behaviour and intention are used as endogenous variables against the predictors of social parameters. The log odds of the age group estimates are positive in reference to the age group 60–65. Thus, the hypothesis that age has a positive influence was confirmed. In addition, although the log odds of younger aged seniors are less negative than those of their senior aged peers, they are significant. This implies that younger older adults buy things online more frequently than their older peers. Furthermore, we found no support for the impact of gender on the behaviour of older people. Furthermore, all educational levels had a positive log odds impact on online shopping behaviour and intention except for the secondary education level (10th class), thus confirming the hypothesis on the impact of education. However, when examined in more detail by log odds weighting, people with higher education levels were more likely to have positive online shopping behaviour and intention than those without, further strengthening the hypothesis. In addition, the log odds of people residing in an urban area compared with rural areas has a highly significant odds increase of 17.62, making it one of the most significant influencers of online shopping behaviour in our research. The opposite is true for people living in rural areas with a log odd of −18.57. This indicates that living in rural areas negatively influences older adults’ intention to shop online and their actual shopping behaviour of older adults (Table 6).

**Table 6:** Direct influence of social factors using generalised linear model.

	Estimate	Std. Error	Z Value	Pr(>|z|)	Sig.
Gender					
Gender Female	-1.1756	0.1618	-7.266	3.69E-13	^***^
Gender Male	-0.3686	0.3001	-1.228	0.219	
Age					
Age60-65	-0.09531	0.21847	-0.436	0.66264	
Age66-70	-0.75911	0.34497	-2.201	0.027771	^*^
Age71-75	-1.75707	0.43856	-4.006	6.16E-05	^***^
Age76-79	-3.93004	1.03227	-3.807	0.000141	^***^
Age80-85	-18.4708	1087.106	-0.017	0.986444	
Age86 and Older	-18.4708	1581.972	-0.012	0.990684	
Education					
Educational Level Basic education (8th class)	-18.57	1103	-0.017	0.987	
Educational Level Initial Vocational Education	16.08	1103	0.015	0.988	
Educational Level Postgraduate University Education	18.16	1103	0.016	0.987	
Educational Level Secondary education (10th class)	-2E-08	1638	0.000	1.000	
Educational Level Secondary specialist education	16.68	1103	0.015	0.988	
Educational Level University education	18.21	1103	0.017	0.987	
Place of Residence					
Place of Residence Rural Area	-18.57	763.42	-0.024	0.981	
Place of Residence Urban Area	17.62	763.42	0.023	0.982	

The SEM model to test the positive impact of social influence on the intention of older people to shop online and actual shopping behaviour were both significant (Table 7). This means there was a standardised score increase for the latent factor. Social Influence is associated with an 0.603 standardised score increase in intention to shop online, and a standardised score increase of 0.188 in the actual shopping behaviour of older people. Furthermore, the health needs of older people were also found to increase their intention to shop online (0.492 standardised score increase), and their actual shopping behaviour (0.294 standardised score increase), both of which are also significant.

**Table 7:** Regression results of structural equation model.

	Estimate	Std. Err	z-value	P(>|z|)	Std.lv	Std. all
SI						
ITSO	0.563	0.08	7.061	0.000	0.603	0.603
ASB	0.204	0.093	2.184	0.029	0.188	0.188
HN						
ITSO	0.405	0.066	6.111	0.000	0.492	0.492
ASB	0.281	0.091	3.085	0.002	0.294	0.294
PU						
ITSO	0.598	0.06	9.974	0.000	0.609	0.609
ASB	0.4	0.066	6.09	0.000	0.35	0.35
PEOU						
ITSO	0.481	0.075	6.429	0.000	0.478	0.478
ASB	0.5	0.078	6.427	0.000	0.428	0.428
FC						
ITSO	0.685	0.067	10.161	0.000	0.72	0.72
ASB	0.252	0.065	3.863	0.000	0.227	0.227
PTAR						
ITSO	0.753	0.05	15.081	0.000	0.789	0.789
ASB	0.188	0.058	3.256	0.001	0.169	0.169

Std. Err =  Standard error. Std. lv indicates that only the latent variables are standardized. Std. all indicates a complete standardized solution whereby both the latent and observed variables are standardized.

Perceived usefulness was significant with a standardised score increase of 0.609 and 0.350 for the intention to shop online and actual shopping behaviour, respectively. In addition, perceived ease of use resulted in a significant standardised score increase of 0.478 in intention to shop online and a significant 0.428 in the actual shopping behaviour. In addition, facilitating conditions had a very significant standardised score increase in intention to shop online (0.720) and a significant influence on actual shopping behaviour. Finally, perceived trust and risk significantly influenced the intention to shop online and the actual shopping behaviour of older adults with a standardised score increase in the latent factors of 0.789 and 0.169, respectively.

The R-squared result in Table 8 shows 87.3% variation in the intention to shop and actual online shopping behaviour were explained by perceived trust and risk. Moreover, it was 84.1% for facilitating conditions, 84.6% for perceived usefulness, 56.8% for health needs, and 75% for perceived ease of use. Meanwhile, Table 9 shows the results of our hypotheses testing. From Table 9, it can be seen that all our hypotheses were supported except for the influence of gender.

**Table 8:** R-squared of the structural equation variables.

Factors	Value (%)
PTAR	0.873
FC	0.841
PU	0.846
HN	0.568
PEOU	0.75

**Table 9:** Hypotheses testing results.

Hypothesis	Relationship	Results
H1_(a)_	Age --> ITSO & ASB	Supported
H1(b)	Gender --> ITSO & ASB	Not supported
H2(a)	Education --> ITSO & ASB	Supported
H2(b)	Higher education --> ITSO & ASB	Supported
H3(a)	POR --> ITSO & ASB	Supported
H3(b)	Rural> Urban	Supported
H4	SI --> ITSO & ASB	Supported
H5	HN --> ITSO & ASB	Supported
H6	PU & PEOU --> ITSO & ASB	Supported
H7	FC --> ITSO & ASB	Supported
H8	PTAR --> ITSO & ASB	Supported

## Discussion and conclusion

### Theoretical contributions

The UTAUT model [18,29] has been well deliberated in recent decades, and extensions of the model have been present in most areas of IT research. Nevertheless, there remains a significant gap in studies related to older people’s online shopping behaviour. Prior research on this group has mostly concentrated on health-related or assistive technology for older people, a stream of research that is broadly referred to as gerontechnology [73]. Therefore, this study makes a number of important theoretical contributions. First, we heed the call of prior studies [10] on the need for more context in research on older people’s shopping behaviour by introducing a conceptual model based on components of the UTAUT model to provide a more holistic investigation of older people’s behaviour. Our model achieved desirable fit and accounted for a cumulative variance of 86%, exceeding that of the original UTAUT model and many extensions of the model [73].

Second, we augment the UTAUT model by introducing four new constructs to provide a more context-specific investigation of older people’s behaviour in online shopping. First, we introduce the concept of older people’s health needs as a motivator or inhibitor of their online shopping behaviour. Drawing from research during and after the Covid-19 pandemic [67], we argue that older people might be more willing to engage in online shopping if their health conditions encouraged or necessitated their usage of online shopping. We found a significant positive relationship between the health needs of older people and their decision to shop online. Although, to the best of our knowledge, the health needs of older people have not been examined in prior studies on online shopping, our findings are consistent with previous studies on the impact of health—including cognition, mobility, vision, and mental health—on the overall internet and technology usage of older people [85].

Furthermore, this paper also extends the UTAUT model with the constructs of perceived trust and risk for the first time in a study of older people engaged in online shopping. Consistent with previous findings [20,21,73,81], we found that trust in online/digital services and the risk associated with them significantly influences the behaviour of older people. In addition, the constructs of educational level and place of residence/settlement are introduced alongside the UTAUT model. While prior extensions of UTAUT have examined the influence of education to some extent, research on the influence of place of residence for older people remains scant. Our findings indicate that both level of education and place of residence influence the behaviour of older people significantly. Place of residence in particular was one of the most significant influencers of older people’s behaviour, as rural residents were far less likely to shop online than people living in urban areas (Table 6). Prior studies had reported inconsistent findings on the rural-urban divide in online shopping [44,47]; however, our findings indicate that the entrenched levels of digital inequality among older people [86] are likely to be exacerbated by living in rural areas.

Finally, contrary to our hypothesis, we found no support for the direct influence of gender in our analysis. Since digital literacy—knowledge of IT products/services—is a significant reason for older people’s low participation in online shopping [67,87], our findings revealed that the socio-demographic factors that potentially influenced older people’s digital literacy, such as their sub-generation (age) and level of education, are stronger factors in explaining their behaviour than gender.

### Practical implications

Many practical implications can be drawn from our study. First, the crucial nature of online shopping and the overall digital economy to the functioning of modern societies means there must be a concerted effort to attract older people to use them. Because older people face structural levels of digital inequality, this effort cannot be left to private businesses alone and must include public policy support. For example, incentives can be offered to businesses to design more older-friendly interfaces for shopping apps and websites. Similarly, marketing campaigns, including word-of-mouth marketing, should be launched to target older customers.

Furthermore, our results on older people’s perceptions of trust and risk shows that it is necessary to provide additional layers of customer protection and security for older people when shopping online. This can include stronger levels of product and seller verification in online marketplaces and a built-in system to make it easier for older people to report online crime. Studies have shown that seniors report cybercrime less than other demographic groups [88]. To overcome this challenge, companies can provide dedicated staff, a helpline, and an easier online system to ensure the process of reporting fraud is easy and comfortable for older people. Marketers and advertisers should also communicate the presence of a safer online shopping experience for older people.

Providing and communicating potential health benefits for older people when they shop online can also increase their online participation. This can include highlighting the benefits of online shopping for older adults with mobility challenges and providing stronger support for older people with vision and cognitive problems. In addition, as the move towards less urban locales grows in many countries, companies, particularly small and medium-sized businesses should invest in improving last-mile delivery services to sub-urban and rural areas.

### Limitations and future research directions

In spite of the social, theoretical, and practical contributions of this research, it is not without limitations. First, this study is based on a cross-sectional survey of a single country. We recommend that future studies test the validity or accuracy of our model in other study locations. In addition, the socio-cultural contexts of the survey participants should be considered when interpreting our findings. Future studies can consider longitudinal study designs, cross-country similarities/differences in older people’s online shopping behaviour, and the role of prior experience, all of which were not considered in the present study. Furthermore, our results on the negative impact of living in rural areas on older people’s online shopping behaviour, and the mixed results of prior studies necessitate further exploration of the topic. More studies are needed to investigate the precise antecedents of older people’s behaviour in rural settings.

In addition, our study includes more socio-demographic factors than previous UTAUT models. However, it does not include a cultural examination of the study subjects. As such, future studies can consider including Hofstede cultural dimensions to this model or other extensions of UTAUT to provide a richer cultural understanding of older people’s online behaviour.

There is also a need for methodological diversity in studies on online shopping, especially for older people. While quantitative surveys using UTAUT, the Technology Acceptance Model (TAM), the Theory of Planned Behaviour (TPB), and other models have grown exponentially in recent decades, qualitative studies have lagged. We believe the structural inequalities older people face in online shopping—and indeed in the overall digital economy—may require more qualitative approaches to allow for a deeper understanding of their challenges. Therefore, future studies should consider methodologies that provide a more inclusive understanding of the social, political, cultural, and environmental factors shaping older people’s online shopping behaviour. This includes discursive analytic, ethnographic, and in-depth interviews.

## Supporting information

S1 Table Results of factor identification and cumulative variance. (DOCX)

S1 Text Survey questionnaire in original language (Russian). (PDF)

S2 Text English translation of survey questions. (PDF)

S3 Text Inclusivity in global research. (DOCX)
