# Supplementary material for: Ageing and digital shopping: Measurement and validation of an innovative framework
Source: PLoS One. 2025 Mar 19;20(3):e0315125. doi: 10.1371/journal.pone.0315125 (PMC11922217; doi:10.1371/journal.pone.0315125)
Supplement: S1 Table — (DOCX) [file pone.0315125.s001.docx]

**Table A:** Factor identification

|  | **Item** | **ML5** | **ML3** | **ML1** | **ML6** | **ML8** | **ML2** | **ML4 ML7** | **h2** | **u2** | **com** |
| --- | --- | --- | --- | --- | --- | --- | --- | --- | --- | --- | --- |
| SI1 | 5 | 0.95 |  |  |  |  |  |  | 0.92 | 0.08 | 1 |
| SI3 | 7 | 0.87 |  |  |  |  |  |  | 0.83 | 0.169 | 1 |
| SI2 | 6 | 0.84 |  |  |  |  |  |  | 0.89 | 0.11 | 1.1 |
| ITSO1 | 8 |  | 0.92 |  |  |  |  |  | 0.97 | 0.031 | 1 |
| ITSO2 | 9 |  | 0.91 |  |  |  |  |  | 0.97 | 0.029 | 1 |
| PTAR1 | 1 |  | 0.3 |  |  |  |  |  | 0.85 | 0.15 | 3.9 |
| ASB1 | 16 |  |  |  |  |  |  |  | 0.76 | 0.24 | 3.4 |
| PEOU3 | 21 |  |  | 0.99 |  |  |  |  | 1 | 0.005 | 1 |
| PEOU2 | 20 |  |  | 0.66 |  |  |  |  | 0.92 | 0.077 | 1.2 |
| PEOU4 | 22 |  |  | 0.61 |  |  |  |  | 0.88 | 0.121 | 1.3 |
| PTAR4 | 4 |  |  |  |  |  |  |  | 0.36 | 0.64 | 6 |
| PU1 | 13 |  |  |  | 0.78 |  |  |  | 0.96 | 0.036 | 1.1 |
| PU2 | 14 |  |  |  | 0.65 |  |  |  | 0.86 | 0.136 | 1.3 |
| PU3 | 15 |  |  |  | 0.38 |  |  |  | 0.85 | 0.149 | 2.8 |
| FC1 | 10 |  |  |  |  | 0.6 |  |  | 0.71 | 0.286 | 1.3 |
| FC3 | 12 |  |  |  |  | 0.53 |  |  | 0.76 | 0.239 | 1.6 |
| FC2 | 11 |  |  |  |  | 0.38 |  |  | 0.88 | 0.125 | 3.3 |
| HN1 | 17 |  |  |  |  |  | 0.98 |  | 1 | 0.005 | 1 |
| HN2 | 18 |  |  |  |  |  | 0.46 |  | 0.74 | 0.263 | 2.5 |
| PEOU1 | 19 |  |  |  |  |  |  | 0.67 | 1 | 0.005 | 1.2 |
| PTAR3 | 3 |  |  |  |  |  |  | 0.54 | 0.91 | 0.086 | 1.5 |
| PTAR2 | 2 |  | 0.37 |  |  |  |  | 0.41 | 0.92 | 0.078 | 2.5 |

Note: The Most significant factor is Social Influence. Also, all communalities $h_{2}$ of the items are acceptable. $u_{2}$ is equivalent to$1-h_{2}$, this means factors unexplained, while $com$is the algebraic sum of $h_{2} and u_{2}$ . Additionally, the PTAR4 question: *I find it risky to share my personal and financial information with online shopping companies*, had communalities below 0.5 and was subsequently eliminated from the model.

**Table B:** Total Cumulative value of the factors

|  | ML5 | ML3 | ML1 | ML6 | ML8 | ML2 | ML4 | ML7 |
| --- | --- | --- | --- | --- | --- | --- | --- | --- |
| Cumulative Variance | 0.16 | 0.31 | 0.46 | 0.57 | 0.66 | 0.73 | 0.8 | 0.86 |

The cumulative of the total variance explained for all the factors is 0.86, which is desired.
