# Supplementary material for: Ageing and digital shopping: Measurement and validation of an innovative framework
Source: PLoS One. 2025 Mar 19;20(3):e0315125. doi: 10.1371/journal.pone.0315125 (PMC11922217; doi:10.1371/journal.pone.0315125)
Supplement: S1 Text — (PDF) [file pone.0315125.s002.pdf]

Старение и покупки в Интернете

Пол
- ☐ Мужской
- ☐ Женский
- ☐ Не скажу

Уровень образования
- ☐ Основное образование (8 классов)
- ☐ Среднее образование (10 классов)
- ☐ Начальное профессиональное образование
- ☐ Среднее специальное образование
- ☐ Высшее образование
- ☐ Ученая степень

Возрастная группа
- ☐ 60 – 65
- ☐ 66 – 70
- ☐ 71 – 75
- ☐ 76 – 80
- ☐ 81 – 85
- ☐ 86 и старше

Я живу в
- ☐ Сельской местности
- ☐ Городе

Вы согласны со следующими утверждениями?

Покупки в Интернете удобны для меня
- ☐ Не согласен
- ☐ Скорее нет, чем да
- ☐ Затрудняюсь ответить
- ☐ Скорее да, чем нет
- ☐ Совершенно согласен

Онлайн-шопинг позволяет мне делать покупки быстрее
- ☐ Не согласен
- ☐ Скорее нет, чем да
- ☐ Затрудняюсь ответить
- ☐ Скорее да, чем нет
- ☐ Совершенно согласен

Покупки через интернет для меня более выгодны
- ☐ Не согласен
- ☐ Скорее нет, чем да
- ☐ Затрудняюсь ответить
- ☐ Скорее да, чем нет
- ☐ Совершенно согласен

Я знаю, как делать покупки в интернете, и для меня это не составляет труда
- ☐ Не согласен
- ☐ Скорее нет, чем да
- ☐ Затрудняюсь ответить
- ☐ Скорее да, чем нет
- ☐ Совершенно согласен

Я легко понимаю функционал онлайн-магазинов и их приложений
- ☐ Не согласен
- ☐ Скорее нет, чем да
- ☐ Затрудняюсь ответить
- ☐ Скорее да, чем нет
- ☐ Совершенно согласен

Я легко пользуюсь онлайн-магазинами и их мобильными приложениями
- ☐ Не согласен
- ☐ Скорее нет, чем да
- ☐ Затрудняюсь ответить
- ☐ Скорее да, чем нет
- ☐ Совершенно согласен

Я легко научился(-лась) делать покупки в интернете
- ☐ Не согласен
- ☐ Скорее нет, чем да

- ☐ Затрудняюсь ответить
- ☐ Скорее да, чем нет
- ☐ Совершенно согласен

Мое состояние здоровья способствуют тому, что я делаю покупки в Интернете
- ☐ Не согласен
- ☐ Скорее нет, чем да
- ☐ Затрудняюсь ответить
- ☐ Скорее да, чем нет
- ☐ Совершенно согласен

Интернет-магазины и приложения разработаны так, что они соответствуют моим ограничениям по здоровью
- ☐ Не согласен
- ☐ Скорее нет, чем да
- ☐ Затрудняюсь ответить
- ☐ Скорее да, чем нет
- ☐ Совершенно согласен

Мои близкие считают, что я должен(-а) делать покупки в интернете
- ☐ Не согласен
- ☐ Скорее нет, чем да
- ☐ Затрудняюсь ответить
- ☐ Скорее да, чем нет
- ☐ Совершенно согласен

Люди, чье мнение мне важно, считают, что я должен(-а) делать покупки в интернете
- ☐ Не согласен
- ☐ Скорее нет, чем да
- ☐ Затрудняюсь ответить
- ☐ Скорее да, чем нет
- ☐ Совершенно согласен

Окружающие предпочитают, чтобы я делал(-а) покупки в интернете
- ☐ Не согласен
- ☐ Скорее нет, чем да
- ☐ Затрудняюсь ответить
- ☐ Скорее да, чем нет
- ☐ Совершенно согласен

У меня есть телефон или компьютер, чтобы делать покупки в интернете
- ☐ Не согласен
- ☐ Скорее нет, чем да
- ☐ Затрудняюсь ответить
- ☐ Скорее да, чем нет
- ☐ Совершенно согласен

У меня есть знания или навыки для совершения покупок в интернете
- ☐ Не согласен
- ☐ Скорее нет, чем да
- ☐ Затрудняюсь ответить
- ☐ Скорее да, чем нет
- ☐ Совершенно согласен

Я могу обратиться за помощью к кому-либо, если у меня возникают проблемы с покупками в интернете
- ☐ Не согласен
- ☐ Скорее нет, чем да
- ☐ Затрудняюсь ответить
- ☐ Скорее да, чем нет
- ☐ Совершенно согласен

Я доверяю известным мне интернет-магазинам
- ☐ Не согласен
- ☐ Скорее нет, чем да
- ☐ Затрудняюсь ответить
- ☐ Скорее да, чем нет
- ☐ Совершенно согласен

Интернет-магазины выполняют свои обязательства и обещания
- ☐ Не согласен
- ☐ Скорее нет, чем да
- ☐ Затрудняюсь ответить
- ☐ Скорее да, чем нет
- ☐ Совершенно согласен

Я верю, что товары, которые я покупаю онлайн, не подделка
- ☐ Не согласен
- ☐ Скорее нет, чем да
- ☐ Затрудняюсь ответить
- ☐ Скорее да, чем нет
- ☐ Совершенно согласен

Я считаю, что делиться своей личной и финансовой информацией с интернет-магазинами - это рискованно
- ☐ Не согласен
- ☐ Скорее нет, чем да
- ☐ Затрудняюсь ответить
- ☐ Скорее да, чем нет
- ☐ Совершенно согласен

Как часто вы совершаете онлайн-покупки в течение месяца?
- ☐ Никогда
- ☐ Редко
- ☐ Иногда
- ☐ Часто
- ☐ Всегда

Я намерен(-а) совершать покупки через интернет в будущем
- ☐ Не согласен
- ☐ Скорее нет, чем да
- ☐ Затрудняюсь ответить
- ☐ Скорее да, чем нет
- ☐ Совершенно согласен

Я планирую продолжать делать покупки в интернете в будущем
- ☐ Не согласен
- ☐ Скорее нет, чем да
- ☐ Затрудняюсь ответить
- ☐ Скорее да, чем нет
- ☐ Совершенно согласен
