# Supplementary material for: Ageing and digital shopping: Measurement and validation of an innovative framework
Source: PLoS One. 2025 Mar 19;20(3):e0315125. doi: 10.1371/journal.pone.0315125 (PMC11922217; doi:10.1371/journal.pone.0315125)
Supplement: S2 Text — (PDF) [file pone.0315125.s003.pdf]

Ageing and Digital Shopping

Gender
- ☐ Man
- ☐ Woman
- ☐ Prefer not to say

Level of Education
- ☐ Basic education (8th class)
- ☐ Secondary education (10th class)
- ☐ Initial vocational education
- ☐ Secondary specialist education
- ☐ University education
- ☐ Postgraduate university education

Age group
- ☐ 60 – 65
- ☐ 66 – 70
- ☐ 71 – 75
- ☐ 76 – 80
- ☐ 81 – 85
- ☐ 86 and older

I live in
- ☐ Rural area
- ☐ Urban area

Do you agree with the following statements?

Shopping online is useful for me
- ☐ Strongly disagree
- ☐ Disagree
- ☐ Neither agree nor disagree
- ☐ Agree
- ☐ Strongly agree

Shopping online allows me to buy things more quickly
- ☐ Strongly disagree
- ☐ Disagree
- ☐ Neither agree nor disagree
- ☐ Agree
- ☐ Strongly agree

Shopping online is more beneficial for me
- ☐ Strongly disagree
- ☐ Disagree
- ☐ Neither agree nor disagree
- ☐ Agree
- ☐ Strongly agree

I know how to shop online and it is easy for me
- ☐ Strongly disagree
- ☐ Disagree
- ☐ Neither agree nor disagree
- ☐ Agree
- ☐ Strongly agree

Shopping websites and apps are clear and understandable
- ☐ Strongly disagree
- ☐ Disagree
- ☐ Neither agree nor disagree
- ☐ Agree
- ☐ Strongly agree

Shopping websites and apps are easy to use for me
- ☐ Strongly disagree
- ☐ Disagree
- ☐ Neither agree nor disagree
- ☐ Agree
- ☐ Strongly agree

It is easy for me to learn to shop online
- ☐ Strongly disagree
- ☐ Disagree

- ☐ Neither agree nor disagree
- ☐ Agree
- ☐ Strongly agree

My health condition is a reason I shop online
- ☐ Strongly disagree
- ☐ Disagree
- ☐ Neither agree nor disagree
- ☐ Agree
- ☐ Strongly agree

Shopping websites or apps support my health needs
- ☐ Strongly disagree
- ☐ Disagree
- ☐ Neither agree nor disagree
- ☐ Agree
- ☐ Strongly agree

My loved ones think that I should shop online
- ☐ Strongly disagree
- ☐ Disagree
- ☐ Neither agree nor disagree
- ☐ Agree
- ☐ Strongly agree

People who influence my behaviour think that I should shop online
- ☐ Strongly disagree
- ☐ Disagree
- ☐ Neither agree nor disagree
- ☐ Agree
- ☐ Strongly agree

People around me prefer that I shop online
- ☐ Strongly disagree
- ☐ Disagree
- ☐ Neither agree nor disagree
- ☐ Agree
- ☐ Strongly agree

I have a phone or computer for shopping online
- ☐ Strongly disagree
- ☐ Disagree
- ☐ Neither agree nor disagree
- ☐ Agree
- ☐ Strongly agree

I have the knowledge or skill for shopping online
- ☐ Strongly disagree
- ☐ Disagree
- ☐ Neither agree nor disagree
- ☐ Agree
- ☐ Strongly agree

I can get help from others if I have problems with shopping online
- ☐ Strongly disagree
- ☐ Disagree
- ☐ Neither agree nor disagree
- ☐ Agree
- ☐ Strongly agree

I trust the online shopping companies I know
- ☐ Strongly disagree

☐ Disagree
☐ Neither agree nor disagree
☐ Agree
☐ Strongly agree

Online shopping companies keep their promises and commitments

☐ Strongly disagree
☐ Disagree
☐ Neither agree nor disagree
☐ Agree
☐ Strongly agree

I believe in the authenticity of products I buy online

☐ Strongly disagree
☐ Disagree
☐ Neither agree nor disagree
☐ Agree
☐ Strongly agree

I find it risky to share my personal and financial information with online shopping companies

☐ Strongly disagree
☐ Disagree
☐ Neither agree nor disagree
☐ Agree
☐ Strongly agree

How often do you shop online every month?

☐ Never
☐ Rarely
☐ Sometimes
☐ Often
☐ Always

I intend to shop online in the future

☐ Strongly disagree
☐ Disagree
☐ Neither agree nor disagree
☐ Agree
☐ Strongly agree

I plan to continue shopping online in the future

☐ Strongly disagree
☐ Disagree
☐ Neither agree nor disagree
☐ Agree
☐ Strongly agree
